# Design of a Novel Six-Axis Wrist Force Sensor

**DOI:** 10.3390/s18093120

**Published:** 2018-09-16

**Authors:** Shanshan Hu, Huaiyang Wang, Yong Wang, Zhengshi Liu

**Affiliations:** School of Mechanical Engineering, Hefei University of Technology, Hefei 230009, China; hushanshan@mail.hfut.edu.cn (S.H.); whuaiyang@mail.hfut.edu.cn (H.W.)

**Keywords:** six-axis wrist force sensor, cross-beams, elastomer designed

## Abstract

A novel elastic body design idea of six-axis wrist force sensor with a floating beam was raised based on the analysis of the robot six-axis wrist force sensor with a floating beam. The design ideas improve the sensor’s dynamic performance significantly, while not reducing its sensitivity. First, the design ideas were described in detail, which were analyzed by mechanical modeling and were verified by finite element analysis. Second, the static simulation analysis of the novel elastomer of sensor was carried out. According to the strain distribution performance, the position of the strain gauges pasted and the connection mode of the full-bridge circuits were decided, which can achieve theoretical decoupling. Finally, the comparison between the static and dynamic performance of the novel sensor and the original sensor with floating beams was done. The results show that the static and dynamic performance of the novel six-axis wrist sensor are all better than the original sensor.

## 1. Introduction

The six-axis wrist force sensor is one of the most important sensors of the robot, which can simultaneously detect the full information of three-dimensional space. It is widely used in position/force control, axle hole matching, contour tracking, coordination of two robots, and other fields of robot [1,2,3,4]. Since piezoelectric elements cannot be used for static force measurement, the commonly used wrist force sensor of the robot is still a strain sensor. Due to the working principle of the strain sensor that the force is measured through its elastic deformation, its dynamic performance is poor compared with that of the piezoelectric sensor. The performance of the strain sensor depends on the design of elastomer first. With the development of robot towards high speed and high precision, the problem of dynamic force measurement is more and more prominent. The six-axis wrist force sensor not only requires high sensitivity in each axis direction and little cross interference, but it also requires adequate work bandwidth to meet the need of dynamic force measurement. Therefore, the core requirements of sensor elastomer design are high sensitivity, good dynamic performance and uncoupled output. The sensitivity reflects the precision of instrument measurement, and the dynamic performance determines the precision and the response time of instrument measurement. Therefore, the sensitivity and dynamic performance are important evaluation criteria of instrument measurement. However, the contradiction between sensitivity and dynamic performance is always a difficulty in elastomer design.

The six-axis wrist force sensor has various elastomers, which have advantages and disadvantages and are suitable for different environments. Its basic structure can be divided into vertical beam type [5], plane beam type [6,7,8], composite beam type [9,10,11], Stewart structure type [12,13], cylinder type [14,15], and so on. The commonly used cross-beam elastomer structure of six-axis wrist force sensor which belongs to plane beam type has the characteristics of small volume, symmetrical, and uncoupled output and integrated processing [16]. So, the cross-beam six-axis wrist force sensor with a floating beam is widely applied in various tasks for robots, and many scholars have been optimizing it [8,17,18] and improving its structure [10,19]. Therefore, the elastomer structure of the six-axis force sensor with floating beam and cross-beams [20] was analyzed in this paper. On the basis of its structure and measuring principle, a novel elastomer structure and strain gauge layout method were designed, trying to ensure a higher sensitivity and have better dynamic performance. Moreover, the design ideas were described in detail, which was analyzed by mechanical modeling and verified by finite element analysis. In the end, comparative analysis was employed with an example to illustrate that the elastomer structure of the novel sensor is superior to the original elastomer structure in both static and dynamic performance.

## 2. Structural Design Ideas of a Novel Six-Axis Wrist Force Sensor

### 2.1. Wrist Force Sensor with Floating Beams

**Figure 1:**
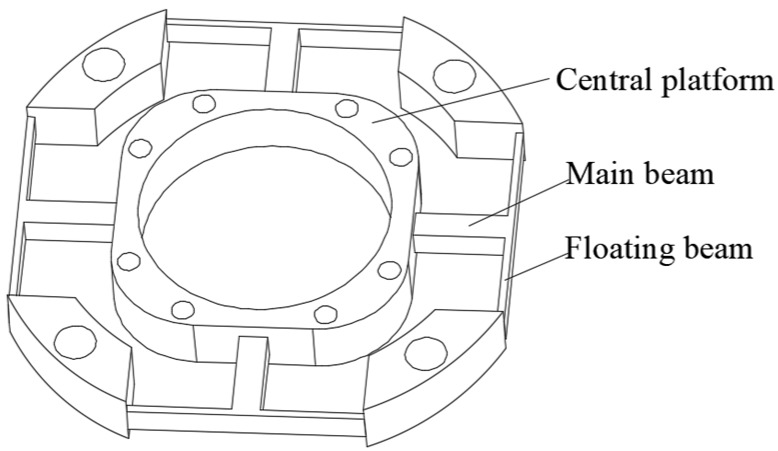
Overall structure of six-axis wrist force sensor.

The elastic body of a wrist force sensor with a floating beam includes the main beams and floating beams. The main beams are a cross structure producing sensitive strain. The floating beams are a thin plate structure which reduce the dimension coupling and isolate strain between dimensions. When the sensor was under a force or moment in one direction, the corresponding strain beam produces bending deflection. Then the force on the sensor can be obtained by use the strain gauges to detection the deformation, after the signal processing and calibration. Figure 1 [16] shows the elastic structure of a six-axis wrist force sensor, which consists of the floating beams, the main beams, and the central platform.

### 2.2. Design Ideas of the Novel Six-Axis Wrist Force Sensor

**Figure 2:**
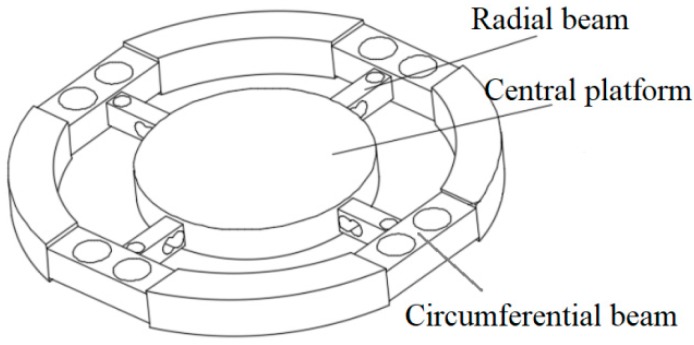
A novel elastomer type of six-axis force sensor.

The novel six-axis force sensor [21] is shown in Figure 2, and the design ideas are as follows:

First, changing the floating beam to H-beam increases the stiffness of the sensor and improves the dynamic performance. Second, drilling holes on the main beam improves the sensitivity of sensor. Third, the part of the main beam near the center platform is processed as a parallel beam to improve the sensor sensitivity. Finally, the strain gauges were pasted at the position of maximum strain on the beam, and the connection mode of the full-bridge circuits is designed so that the sensor can be decoupled theoretically.

Due to the different applications of the six-axis wrist force sensor, the size of the central platform and the outer diameter are not considered here, and the structure and size of the main beams and the floating beams as shown in Figure 1 are only studied. For the convenience of explanation, the direction of the main beams are defined as the X and Y axes, and the Z axis is passed through the center of the central platform. The main beam or the radial beam described below is in the X direction, and the floating beam or the circumferential beam described below is a beam connected to the main beam or the radial beam in the X direction. The static and dynamic performance of the sensor affected by various sizes in this paper refers to [20].

When the force of *F_x_* or *F_y_* is applied to the six-axis wrist force sensor, the strain gauges for measuring the force are arranged on the side of the main beam. In order to obtain a better sensitivity, the main beam needs to get a larger dominant force. In other words, the more the floating beam does not hinder the deformation of the main beam, the better sensitivity it has. So, the height of the floating beam should be as small as possible, and the width should be as narrow as possible. For the main beam, under the same force, the lager the deformation of the place pasted strain gauges, the better. So, the width and the height of the main beam should be as small as possible. Meanwhile, the smaller the sizes, the worse dynamic performance of the sensor, which creates a contradiction between static performance and dynamic performance. Therefore, many people have conducted in-depth research here to try to find the optimal sizes that can satisfy sensitivity and dynamic performance requirements. In this paper, we modified the structure of the sensor, such as changing the floating beam into an H-shaped structure. We call it the circumferential beam as shown in Figure 2. This structure can realize the function of the floating beam, that realizing translation of the X direction and rotation of the Y and Z direction and a bit of translation and rotation in the other directions under force or torque. When the force of *F_x_* or *F_y_* is applied, the stress concentrates on both sides of the hole, and the strain of the Y direction is larger. So, the strain gauges are pasted here for measuring the Y direction strain. In this way, the loading force can be measured more accurately. As for the design of the hole shape, we will discuss it in depth later.

When the force of *F_z_* is applied to the six-axis wrist force sensor, the strain gauges for measuring this force are arranged on the upper and lower surfaces of the main beam. To get better sensitivity of measuring *F_z_*, the width and height of the floating beam should be as small as possible, which make the floating beam realize translation of the X direction and rotation of the Y direction. Similarly, the smaller the better for the width and height of the main beam. However, the dynamic performance will be very bad in this way. Therefore, when design the structure, it is necessary to consider the idea that the strain should be gathered in one place where strain gauges are connected. This idea means that the deformation is mainly concentrate on the position where is used to measure the strain, and the deformation of other positions is very small. In this way, under the same sensitivity of measuring *F_z_*, the dynamic performance of sensor will be better. So, the hole is designed to be punched on the main beam, which concentrates the beam deformation here. Because the strain of measuring *F_z_* on the main beam is in X direction, the hole is punched along the Y axis, as shown in Figure 2. The strain gauges are pasted on the upper and lower surfaces of the hole for measuring the X direction strain.

When loading the torque of M_x_ and M_y_ on the six-axis wrist force sensor, the strain gauges for measuring the moments are arranged on the upper and lower sides of the main beam. The influence of the sizes of the floating beam and main beam to measuring *M_x_*, *M_y_* are similar to the influence of measuring *F_x_*. The design idea is the same too—that the hole is punched along the Y axis, which is connected with the hole that measures the force *F_z_*. The beam with two connected holes forms a parallel beam structure, as shown in Figure 2. The strain gauges are pasted on the upper and lower surfaces of the hole for measuring the X direction strain.

When loading *M_z_* on the six-axis wrist force sensor, the strain gauges for measuring this torque are arranged on both sides of the main beam. The influence of the sizes of the floating beam and main beam to measuring *M_z_* are similar to the influence of measuring *F_x_*. When the torque of *M_z_* is loaded, the strain should be gathered on one place where pasted strain gauges. A hole is punched along the Z axis on the main beam, as shown in Figure 2. The strain gauges are pasted on both sides of the hole for measuring the X direction strain.

## 3. Mechanics Analysis

The above chapter have introduced the design idea of the novel six-axis wrist force sensor. In this chapter, some structure of the designed sensor will be analyzed with mechanics.

### 3.1. Comparison between H-Beam and Floating Beam

The H-beam is defined as an “H” shape beam, which is composed of two single beams connected in the middle. The shape of two single beams is not fixed. Here, for the convenience of calculation, the floating beam is a uniform beam with rectangular cross section, and the H-beam is composed of two single beams the same size as the floating beam.

**Figure 3:**
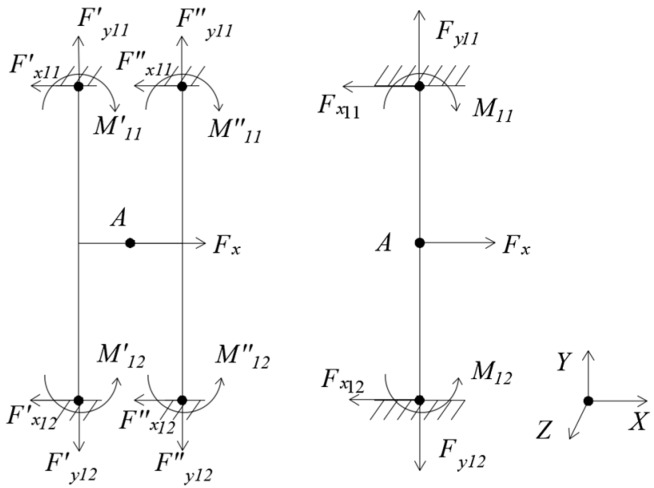
Mechanical models of floating beam and H-beam under *F_x._*

The H-beam and the floating beam in X-axis are taken for mechanical analysis. The H-beam and floating beam have the same length *l*, height *h*, and thickness *t* that also means the width of the beam. The upper and lower ends of the beams are restrained. The displacement *y* and rotation angle *θ* of point A are compared under the same force or torque. The mechanical analysis of H-beam and floating beam under different working conditions are as follows [22]:

(1) Loading *F_x_*, as Figure 3.

Under *F_x_*, the floating beam has only an X-directional displacement $y_{x1}=\frac{F_{x}\cdot l^{3}}{192\cdot EI_{1}}$, and the H-beam has only an X-directional displacement $y_{x1}^{\prime }=\frac{0.5\cdot F_{x}\cdot l^{3}}{192\cdot EI_{1}}$. E is elasticity modulus. The moment of inertia $I_{1}=\frac{h\cdot t^{3}}{12}$. It can be seen that the X-directional displacement of the floating beam is twice that of the H-beam.

**Figure 4:**
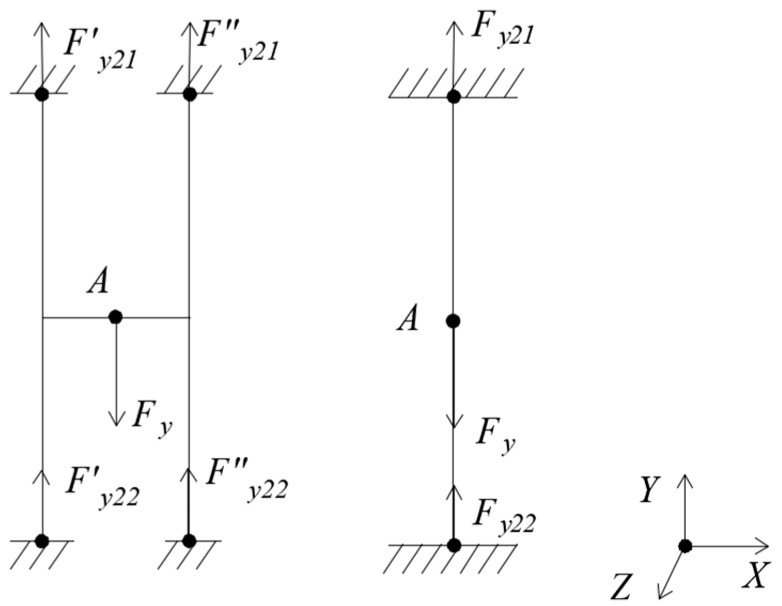
Mechanical models of floating beam and H-beam under *F_y._*

(2) Loading *F_y_*, as Figure 4.

Under *F_y_*, the floating beam has only a Y-directional displacement $y_{y}=\frac{F_{y}\cdot l}{EA}$, and the H-beam only has a Y-directional displacement $y_{y}^{\prime }=\frac{0.5\cdot F_{y}\cdot l}{EA}$. The cross-sectional area A=t⋅h. It can be seen that the Y-directional displacement of floating beam is twice that of the H-beam.

**Figure 5:**
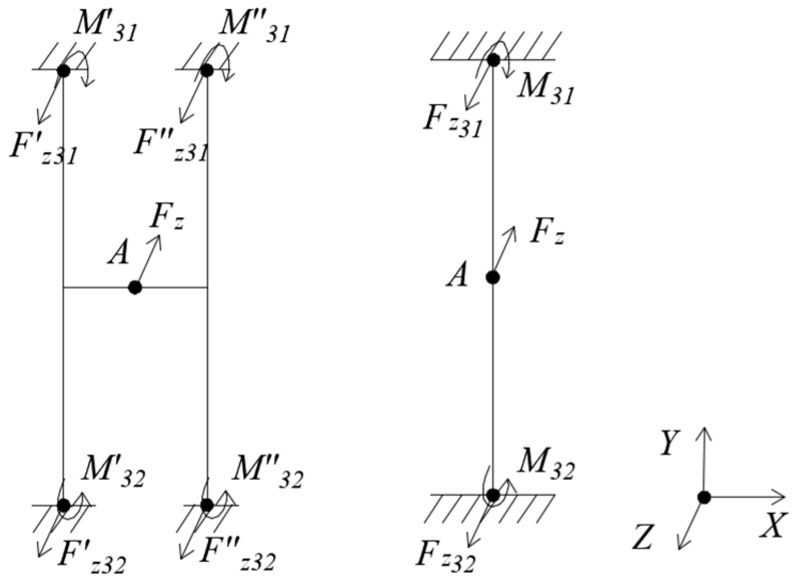
Mechanical models of floating beam and H-beam under *F_z._*

(3) Loading *F_z_*, as Figure 5.

Under *F_z_*, the floating beam has only a Z-directional displacement $y_{z}=\frac{F_{z}\cdot l^{3}}{192\cdot EI_{3}}$, and the H-beam has only an Z-directional displacement $y_{z}^{\prime }=\frac{0.5\cdot F_{z}\cdot l^{3}}{192\cdot EI_{3}}$. The moment of inertia $I_{3}=\frac{t\cdot h^{3}}{12}$. It can be seen that the Z-directional displacement of floating beam is twice that of the H-beam.

**Figure 6:**
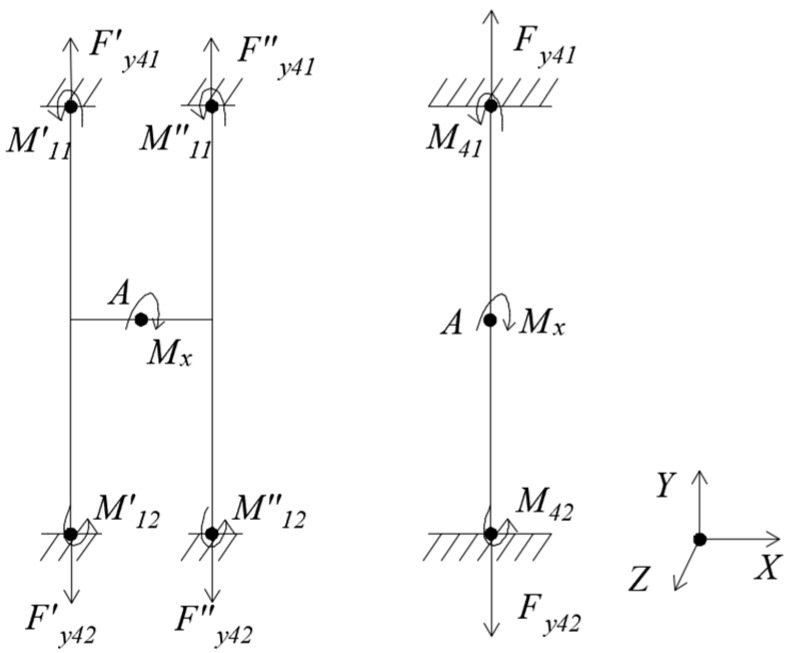
Mechanical models of floating beam and H-beam under *M_x_.*

(4) Loading *M_x_*, as Figure 6.

Under *M_x_*, the floating beam has only a X-directional rotation angle $\theta_{x}=\frac{M_{x}\cdot l}{16\cdot EI_{3}}$, and the H-beam has only an X-directional rotation angle $\theta_{x}^{\prime }=\frac{0.5\cdot M_{x}\cdot l}{16\cdot EI_{3}}$. The moment of inertia $I_{3}=\frac{t\cdot h^{3}}{12}$. It can be seen that the X-directional rotation angle of floating beam is twice that of H-beam.

**Figure 7:**
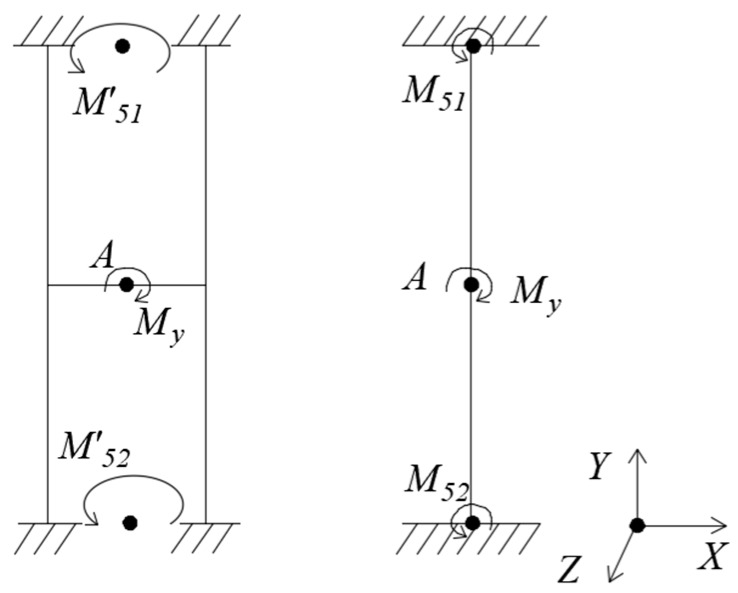
Mechanical models of floating beam and H-beam under *M_y_.*

(5) Loading *M_y_*, as Figure 7.

Under *M_y_*, the floating beam has only a Y-directional rotation angle $\theta_{y}=\frac{M_{y}\cdot l}{4GI_{p1}}$, and the H-beam has only an Y-directional rotation angle $\theta_{y}^{\prime }=\frac{M_{y}\cdot l}{4GI_{p1}^{\prime }}$. G is shear modulus. It can be seen that the torsional moment of inertia *I_p_*_1_ of the floating beam is much smaller than the torsional moment of inertia *I′_p_*_1_ of the H-beam. So, the Y-directional rotation angle of the floating beam is much larger than that of the H-beam.

**Figure 8:**
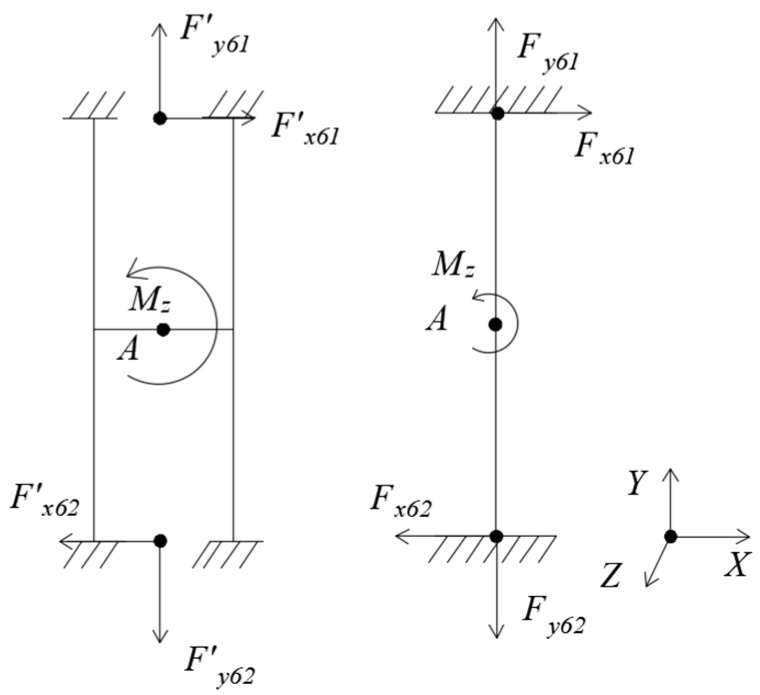
Mechanical models of floating beam and H-beam under *M_z._*

(6) Loading *M_z_*, as Figure 8.

Under *M_z_*, the floating beam has only a Z-directional rotation angle $\theta_{z}=\frac{M_{z}\cdot l}{4GI_{p2}}$, and the H-beam has only an Z-directional rotation angle $\theta_{z}^{\prime }=\frac{M_{z}\cdot l}{4GI_{p2}^{\prime }}$. It can be seen that the torsional moment of inertia *I_p_*_2_ of the floating beam is much smaller than the torsional moment of inertia *I′_p_*_2_ of the H-beam. So, the Z-directional rotation angle of the floating beam is much larger than that of the H-beam.

From the above mechanical analysis, it can be seen that the stiffness of the H-beam is greater than that of the floating beam in all directions. Where the rotational stiffness of Y-axis and Z-axis is much larger than that of the floating beam, and the stiffness of the other four directions is twice that of the floating beam.

As the theoretical model is simplified and the calculation results are complex and inaccurate, the finite element simulation is necessary. We suppose that *l* = 40 mm, *t* = 1.5mm, and the two beams spacing of H-beam *l*_1_ = 7 mm. The material is LY12 aluminum alloy. The upper and lower ends of H-beam and floating beam are fixed. Measuring *F_x_* = 50 N, *F_y_* = 50 N, *F_z_* = 50 N, *M_x_* = 2.5 Nm, *M_y_* = 2.5 Nm, and *M_z_* = 2.5 Nm respectively, the finite element simulation results are shown in Table 1.

It can be seen from Table 1 that the results of the finite element simulation and mechanical analysis are basically the same. So, the above mechanical analysis is correct. After the H-beam is used to replace the floating beam, its stiffness in all directions are improved, especially in the directions of around Y-axis and around Z-axis. However, this change hinders the measurement of force or torque by the main beam. In other word, it reduces the sensor sensitivity. So in the following part, we will introduce a method that punching holes in the main beam to improve the sensitivity and solve the questions about the displacement and rotation angle of H-beam.

### 3.2. Comparison between Single Beam and Parallel Beam

We define that the parallel beam is composed of two single beams that the two ends are connected, and the shape of two single beams are not fixed. Here, for the convenience of calculation, the single beam is a uniform beam with rectangular cross section, and the parallel beam is composed of two uniform single beams with a rectangular cross section.

**Figure 9:**
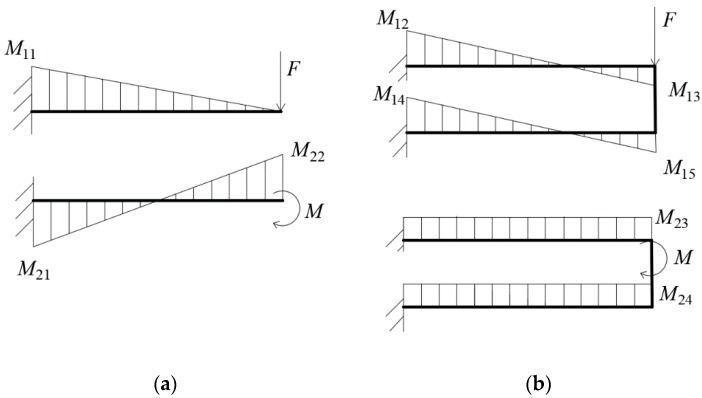
(**a**) Moment diagram of single beam. (**b**) Moment diagram of parallel beam.

It can be seen from Table 1 that the floating beam mainly restricts the degrees of freedom of *y*, *z*, and *θ_x_*. From the perspective of the entire elastomer, when the central plate is subjected to the force of *F_z_* or the moment of *M_y_*, in order to facilitate analysis and simplify calculation, the main beam can be equivalent to a cantilever beam. The force analysis of single beam and parallel beam in the shape of cantilever beam is carried out under the action of *F* force or *M* moment. The moment diagrams are as follows Figure 9a,b [23].

In Figure 9a,b $M_{11}=F\cdot l$, $M_{21}=M_{22}=M$, $M_{12}=M_{14}=\frac{Fl}{2}\cdot \frac{3K+1}{6K+1}$, $M_{13}=M_{15}=-\frac{Fl}{2}\cdot \frac{3K}{6K+1}$, $M_{23}=M_{24}=\frac{M}{2}\cdot \frac{1}{12K+2}$, where *K* is the stiffness ratio between the parallel beam and the vertical beam, *l* is the length of the beam [23].

According to the $\varepsilon =\frac{M\cdot y_{e}}{EI}$, if the section of single beam is rectangle *b*_1_ × *h*_1_, and the section of parallel beam is two rectangles *b*_2_ × *h*_2_, then the moment of inertia $I=\frac{bh^{3}}{12}$, $y_{e}=0.5h$. If K→∞, then $0.25M_{11}=M_{12}=M_{14}=M_{13}=M_{15}$. When $b_{1}=b_{2}$, $h_{2}<0.5h_{1}$, the strain of *F* measured with the parallel beam will be greater than that of the single beam, and $M_{23}=M_{24}\to 0$. Therefore, an appropriate value of *K* should be selected, such as take *K* = 0.01. At this time, $M_{12}=M_{14}=0.4858M_{11}$, $M_{13}=M_{15}=0.01415M_{11}$, $M_{23}=M_{24}=0.2358M_{21}$. From these data, it can be seen that the force of *F* is measured with the place near the fixed end and the moment of *M* is measured with the loading end. When $b_{1}=b_{2}$, $h_{2}<0.48h_{1}$, the strain of *F* measured with the parallel beam will be greater than that with the single beam, and the strain of M measured with the parallel beam will be also greater than that with the single beam. Above all, if an appropriate *K* value can be obtained, the strain measured with parallel beam will be bigger than that with single beam.

## 4. Design of a Novel Six-Axis Wrist Sensor

According to the theory of elastic mechanics [24], the stress around the hole will be much greater than the place that without the hole or far away from the hole. This phenomenon is called ‘outlets stress concentration’. Therefore, after punching a hole in a suitable position of the sensor beams, the stress can be concentrated around the hole, which can improve the sensitivity of measurement.

**Figure 10:**
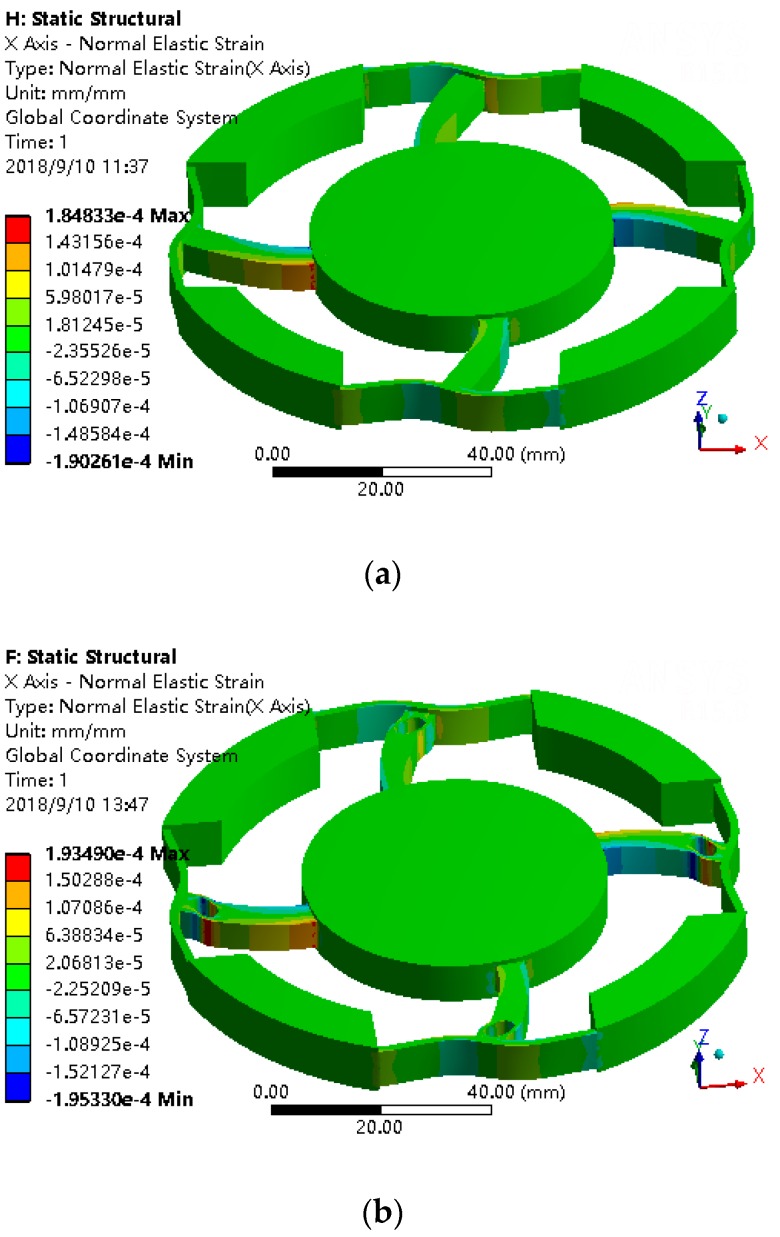
(**a**) The X direction strain without hole under *M_z_* = 2.5 Nm. (**b**) The X direction strain with hole under *M_z_* = 2.5 Nm.

When loading the torque of *M_z_* on the six-axis wrist force sensor, the X-strain diagram is shown in Figure 10a. Drilling a Z-axis through-hole in the main beam, and the strain near the through-hole is used to measure *M_z_*. We positioned the hole near the H-beam temporary. The X-strain diagram is shown in Figure 10b. As seen from Figure 10a,b, when *M_z_* torque is loaded, the strain on the main beam is concentrated on both sides of the holes after drilling, and the holes do not affect the stress deformation of other positions.

**Figure 11:**
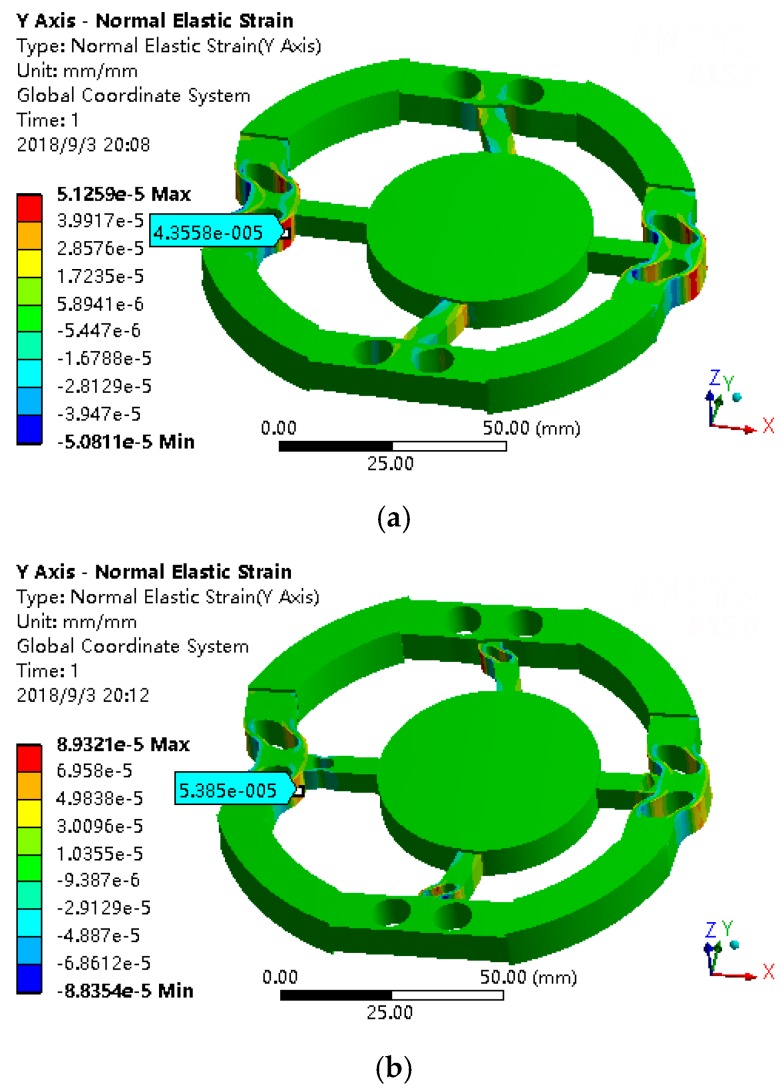
(**a**) The Y direction strain without hole under *F_x_* = 50 N. (**b**) The Y direction strain with hole under *F_x_* = 50 N.

When loading the force of *F_x_* on the six-axis wrist force sensor, the Y-strain diagram is shown in Figure 11. For the convenience of processing, the floating beam is directly drilled two through-holes to form H-beam structure. The floating beam with two through-holes is named after circumferential beam. As can be seen from the Figure 11, the strain is concentrated around the vertical hole of the circumferential beam, which can be used to measure *F_x_*. Therefore, unlike the sensor with a floating beam, the force of *F_x_* is measured with the sides of the hole in the circumferential beam.

If the floating beam is changed into circumferential beam, and loading *F_x_* on the sensor, the Z-axis through-hole of the main beam can be used to solve the problems of smaller angle *θ_x_* of H-beam as discussed earlier and improve the sensitivity of measuring *F_x_*. As shown in Figure 11a,b, when the force of *F_x_* = 50 N is loaded, the strain around the hole of circumferential beam increases from 4.36 × 10^−5^ to 5.39 × 10^−5^. The stress has significantly increased. So, the sensitivity of measuring *F_x_* has improved in this way.

The principle of measuring *F_x_* is the same as measuring *F_y_*. We do not discuss it here.

The sensor with floating beam measures *F_z_*, *M_x_*, and *M_y_* with strain on the upper and lower surface of the main beam. The novel sensor uses the same surface here with the different principle of measurement. Parallel beam structure is adopted here. According to the theoretical analysis of mechanics that mentioned before, the stiffness ratio K between parallel beam and vertical beam is smaller, the higher the sensitivity of *F_z_*, *M_x_*, and *M_y_* measured with elastomer are. The value of K is related to the structural size of the parallel beam. If the parallel beam is thin, the value of K is going to go up, but awful dynamic performance in the Z direction of the parallel beam will appear. Therefore, optimization design is required to improve sensor’s static and dynamic performance simultaneously. Above all, we decide the structure of the main beam as follows: two holes are punched on the main beam, which are located near the central platform. The direction of the hole is perpendicular to the side of the main beam, and the two holes are connected. This structure can be equivalent to the parallel beam structure. The main beam with one vertical hole and one horizontal double hole is named radial beam. According to the above mechanical analysis, *F_z_* is measured with the upper and lower surfaces of the hole that closer to the central platform of the two connected holes. The upper and lower surfaces of another hole are used to measure *M_x_* and *M_y_*.

## 5. Strain Gauges Position and Connection Principles of Circuits

### 5.1. Simulation of the Novel Sensor

By using ANSYS software, the static finite element analysis of the novel wrist force sensor was carried out. The force and torque of six directions were respectively loaded to observe the strain distribution of the sensor elastomer, which provide references for determining the paste location of the strain gauges and summarizing connection mode of the bridges.

#### 5.1.1. Simulation Modeling

We define that the directions of the radial beams of the sensor elastic body are X axis and Y axis directions of the coordinate system, and the Z axis passes through the center of the central platform. The elastomer material is LY12 aluminum alloy, and its characteristic is elasticity modulus E = 7.1 × 10^10^ Pa, Poisson’s ratio *σ* = 0.33, and density *d* = 2.77 × 10^3^ kg/cm^3^. The geometric dimensions of sensor elastic body are shown in Table 2, where *t*_1_ is the minimum thickness beside the vertical hole on the circumferential beam called hole 1, *t*_2_ is the minimum thickness beside the vertical hole on the radial beam called hole 2, *t*_3_ is the minimum thickness beside the hole away from the central platform in the connected holes on the radial beam called hole 3, and *t*_4_ is the minimum thickness beside the hole near the center stage on the radial beam called hole 4.

#### 5.1.2. Simulation Result

**Figure 12:**
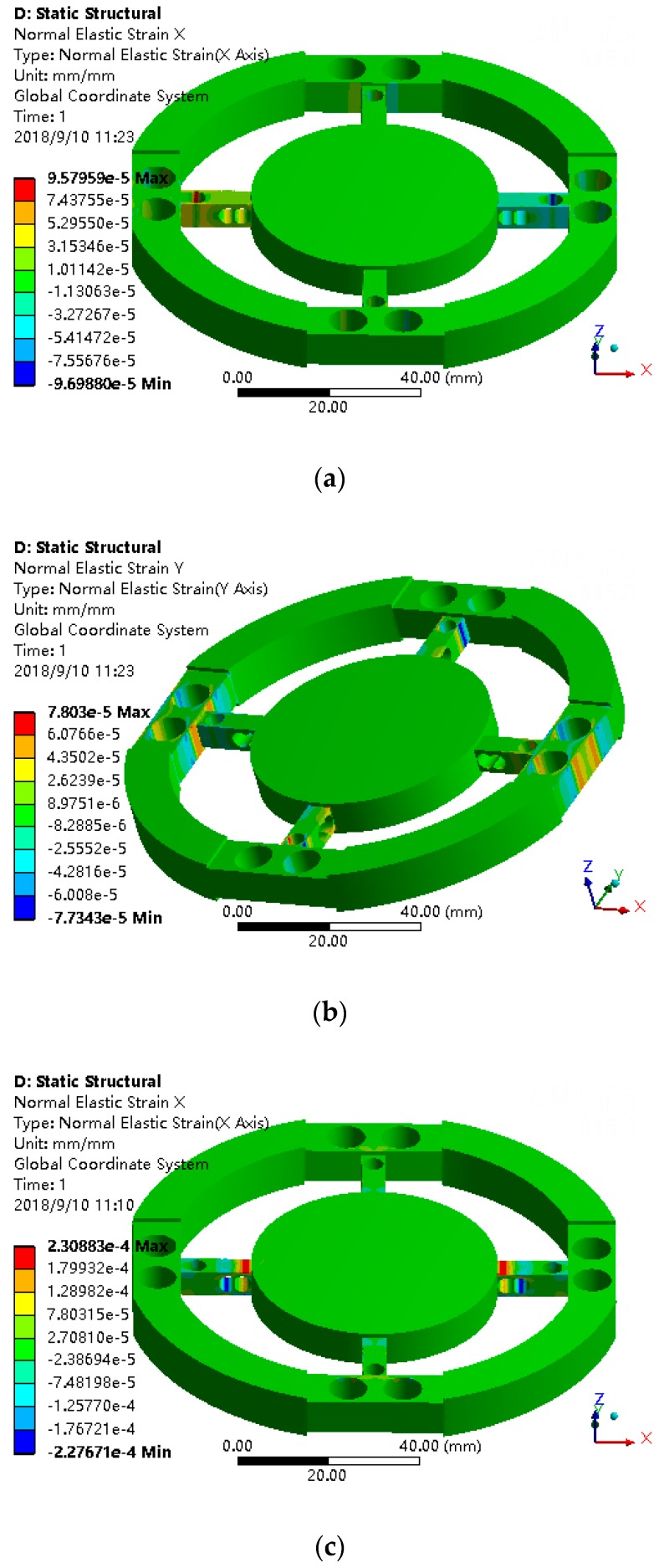

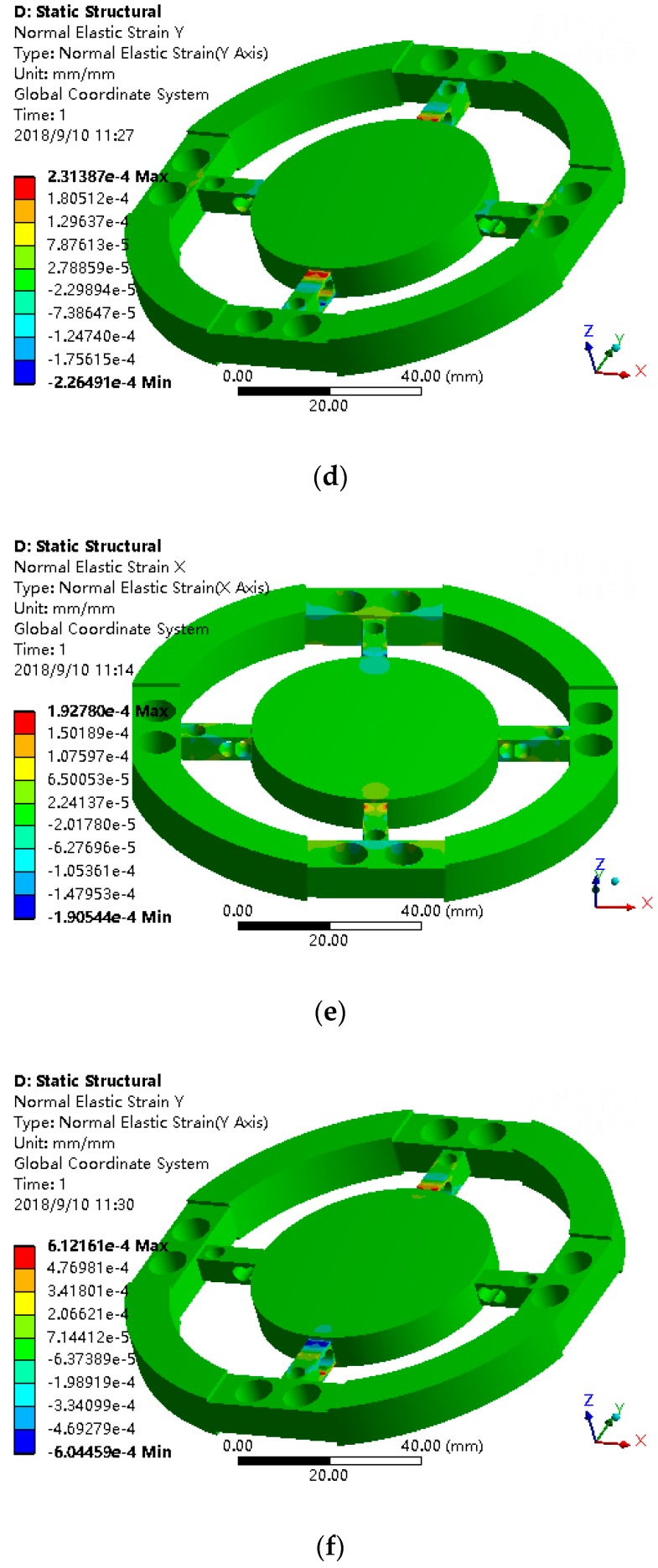

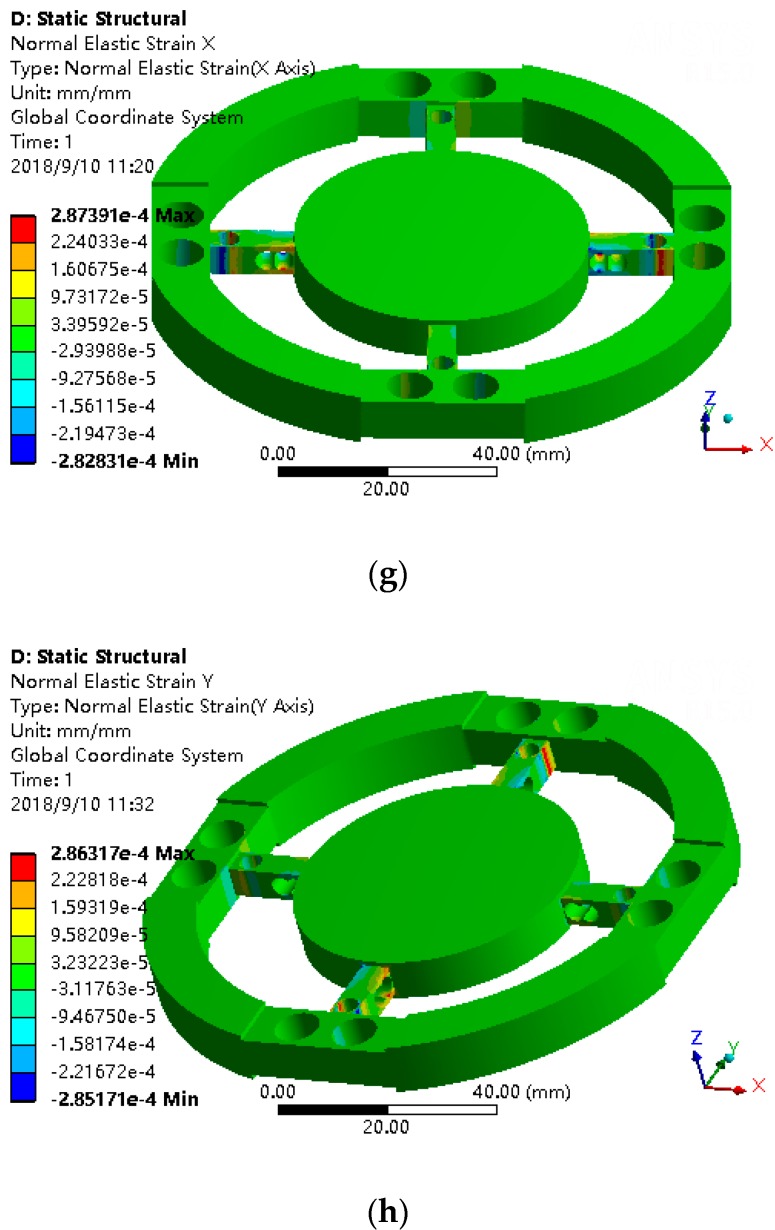
(**a**) The X direction strain under *F_x_* = 50 N. (**b**) The Y direction strain under *F_x_* = 50 N. (**c**) The X direction strain under *F_z_* = 50 N. (**d**) The Y direction strain under *F_z_* = 50 N. (**e**) The X direction strain under *M_x_* = 2.5 N·m. (**f**) The Y direction strain under *M_x_* = 2.5 N·m. (**g**) The X direction strain under *M_z_* = 2.5 N·m. (**h**) The Y direction strain under *M_z_* = 2.5 N·m.

The circumferential support freedoms are completely constrained. Considering the symmetry of the structure and boundary constraints and reducing calculation, the effects of *F_x_*, *F_z_*, *M_x_*, and *M_z_* are only studied here. The output strain of software can be the normal strain, equivalent strain, and so on. Here, according to the strain requirements to be measured, the output strain of the circumferential beam on the X-axis should be Y direction strain. The output strain of the circumferential beam on the Y-axis should be X direction strain. The output strain of radial beam in X direction should be X direction strain. The output strain of the radial beam in the Y-axis direction should be the Y direction strain. After loading *F_x_* = 50 N, *F_z_* = 50 N, *M_x_* = 2.5 N·m, and *M_z_* = 2.5 N·m respectively on the central platform, the strain diagrams are shown in Figure 12.

As can be seen from Figure 12a,b, after loading *F_x_*, one of the X direction radial beams is stretched, another is compressed. The two sides of the Hole 2, Hole 3, and Hole 4 in the X direction radial beams have distinct and symmetrical X direction strain. Compare to one X direction radial beam, the strain of the other beam is equal and opposite in direction. The two sides of the Hole 2 in the Y direction radial beams have distinct Y direction strain which is equal and opposite in direction. Compared to one Y direction radial beam, the strain of the other Y direction radial beam are the same. The two sides of the Hole 1 in the circumferential beam on the X-axis have distinct Y direction strain. The strain beside four holes in the circumferential beam on the X-axis are symmetric about X-axis and equal and opposite about Y-axis.

As can be seen from Figure 12c,d, after loading *F_z_*, the two sides of the Hole 3 and Hole 4 in the X direction radial beams have distinct X direction strain which are equal and opposite in direction. The two sides of the Hole 3 and Hole 4 in the Y direction radial beams have distinct Y direction strain which is equal and opposite in direction. The strain beside Hole 3 and Hole 4 are symmetric about the Z-axis.

As can be seen from Figure 12e,f, after loading *M_x_*, the two sides of Hole 3 and Hole 4 in the Y direction radial beams have distinct Y direction strain which are equal and opposite in direction. The strain beside Hole 3 and Hole 4 are equal and opposite in about the X-axis.

As can be seen from Figure 12g,h, after loading *M_z_*, the two sides of the Hole 2 in the circumferential beams have distinct strain which are symmetric about the origin.

It can be seen from the above simulation results that punching holes in the beams can make the strain concentrate both sides of the holes, which can meet the design requirements of strain concentration.

### 5.2. Strain Gauge Position and Connection Principles of Full-Bridge Circuits

**Figure 13:**
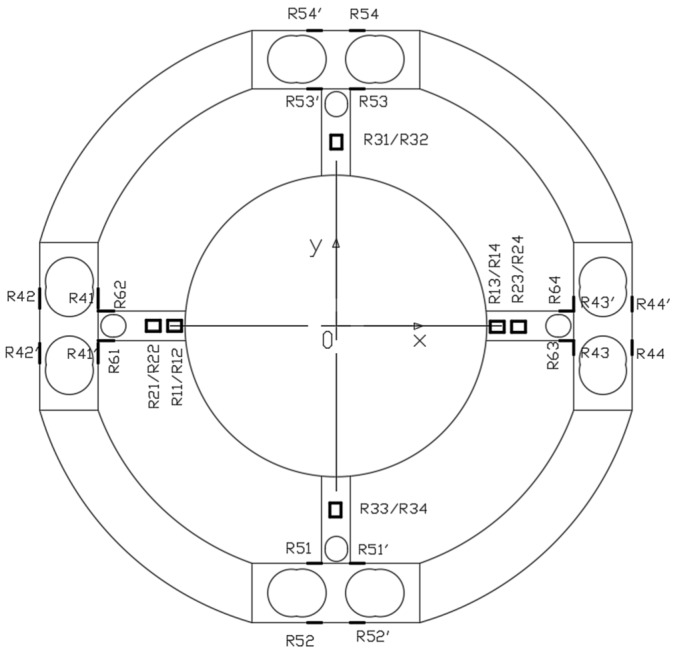
The strain gauges position of the novel six-axis force sensor.

According to the design ideals and the above simulation analysis results, eight strain gauges are pasted on the sides of Hole 1 in the X direction circumferential beams to measure *F_x_*, and eight strain gauges are pasted on the sides of the Hole 1 in the Y direction circumferential beams to measure *F_y_*. Four strain gauges are pasted on the sides of Hole 2 in the X direction radial beams to measure *M_z_*. Four strain gauges are pasted on the sides of the Hole 4 in the X direction radial beams to measure *F_z_*. Four strain gauges are pasted on the sides of Hole 3 in the X direction radial beams to measure *M_y_*. Four strain gauges are pasted on the sides of Hole 3 in the Y direction radial beams to measure *M_x_*. The strain gauge positions are shown in Figure 13.

According to the above simulation results, when loading force or torque on the sensor elastic body, the plus or minus of strain are shown in Table 3. In order to realize decoupling in theory, the connection principles of full-bridge circuits are as follows:

Measuring force *F_x_*: R41 + R41′ − (R42 + R42′) − (R43 + R43′) + R44 + R44′

Measuring force *F_y_*: R51+R51′ − (R52 + R52′) − (R53 + R53′) + R54 + R54′

Measuring force *F_z_*: R11 − R12 + R13 − R14

Measuring torque *M_x_*: −R31 + R32 + R33 − R34

Measuring torque *M_y_*: −R21 + R22 + R23 − R24

Measuring torque *M_z_*: −R61 + R62 + R63 − R64

At this moment, it can be seen from Table 3 that under loading of forces or torques *F_y_*, *F_z_*, *M_x_*, *M_y_*, *M_z_*, the output of the full-bridge circuit measuring *F_x_* is zero, due to the symmetry of the sensor elastic body. The other full-bridge circuits can also perform the same function. Therefore, when measuring six-axis forces and torques, the sensor can be decoupled theoretically.

## 6. Example

**Figure 14:**
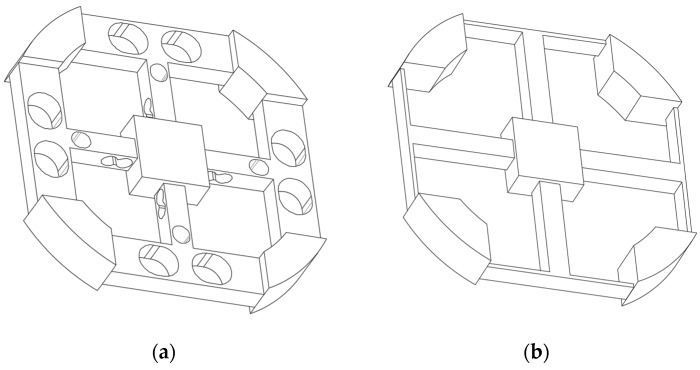
(**a**) Elastic body of Sensor 2. (**b**) Elastic body of Sensor 1.

In order to illustrate the superiority of the novel six-axis wrist force sensor, the novel sensor model is used to compared with the model Example 2 in the paper [8]. In order to express more clearly, the model in the paper [8] is called Sensor 1, and the novel sensor is called Sensor 2. The sizes of Sensor 1 have been determined in the paper. For convenience of comparison, the outer diameter, sizes of center platform, length and height of floating beam, width and height of main beam of Sensor 2 are the same as those of Sensor 1. The dimensions of two sensors are shown in Table 4, and the geometric models are shown in Figure 14.

For comparison, the same simulation software ANSYS, the same constraints, the same materials, and the same load were used for the two sensors in simulation calculation. Two sensor elastomers are respectively loaded with forces *F_x_* = 100 N, *F_z_* = 100 N, torques *M_x_* = 3 N·m, *M_z_* = 3 N·m, and the materials of the elastic body are consistent with the Section 2. The circuits of Sensor 2 are connected according to the Section 3, and the circuits of Sensor 1 are connected according to Figure 2 in paper [8]. After simulation, the total strain measured by the full-bridge circuits are listed in Table 5. The total strain also means the sum strain of the place pasted strain gauges of one full-bridge circuit. The first six orders natural frequencies and the modes of vibration are listed in Table 6.

It can be seen from Table 5 that the sensitivity of six-axis wrist force Sensor 2 measuring *F_x_*, *F_y_*, *M_x_*, and *M_y_* is close to that of Sensor 1, and the sensitivity measuring *F_z_* and *M_z_* is much better than that of Sensor 1. As can be seen from Table 6, the natural frequency of Sensor 2 is much better than Sensor 1 in six directions. Compared with Sensor 1, Sensor 2 can greatly improve dynamic performance without decreasing sensitivity. Above all, the structure of the novel six-axis wrist force sensor is superior to the structure of the original six-axis wrist force sensor in both static and dynamic performance.

In order to compare with Sensor 1, most dimensions of Sensor 2 are the same as Sensor 1. In future studies, we should pursue optimization of the structure of Sensor 2 to achieve better static and dynamic performance.

## 7. Conclusions

(1) This article designs a novel elastomer structure of six-axis wrist force sensor based on the structure of the original six-axis wrist force sensor with cross-beams and floating beams, and described the design ideas in detail. Including changing the floating beams to the H-beams to improve the dynamic performance of sensor and punching holes in beams and using parallel beams structures to increase sensitivity of sensor. In the process of design, the advantages of the structures were analyzed and compared by mechanical modeling and were verified by finite element analysis.
(2) In the process of static simulation analysis, we observe the influence rule of the strain distribution of the novel sensor elastomer under loading various forces and torques, and sum up the best positions where strain gauges are pasted and the connection modes of full-bridge circuits which can achieve decoupling in theory.
(3) After static and dynamic simulation analysis of the two sensor elastomers, the results show that the novel sensor is superior to the original six-axis wrist force sensor with cross-beams and floating beams on both static and dynamic performance, especially on the dynamic performance. The performance of the sensor is improved, which is the base and precondition of the control of the robot with high speed and high precision
(4) The optimization design of this novel six-axis wrist force sensor elastomer should be done, which can further improve its performance to meet the requirements of high-speed and high-precision operation robots.

## Figures and Tables

**Table 1:** Finite element simulation results of H-beam and floating beam

	*F_x_* = 50 N	*F_y_* = 50 N	*F_z_* = 50 N	*M_x_* = 2.5 Nm	*M_y_* = 2.5 Nm	*M_z_* = 2.5 Nm
H-beam	*y_x_* = 3.8 × 10^−2^ mm	*y_y_* = 2.5 × 10^−4^ mm	*y_z_* = 2.5 × 10^−3^ mm	*θ_x_* = 1.2 × 10^−3^	*θ_y_* = 4.8 × 10^−3^	*θ_z_* = 7.6 × 10^−4^
Floating beam	*y_x_* = 7.3 × 10^−2^ mm	*y_y_* = 5.1 × 10^−4^ mm	*y_z_* = 4.9 × 10^−3^ mm	*θ_x_* = 2.2 × 10^−3^	*θ_y_* = 8.5 × 10^−2^	*θ_z_* = 2.5 × 10^−2^

**Table 2:** Basic dimensions of the novel six-axis wrist sensor elastomer

	Length × Width × Height (mm)	Minimum Thickness Beside the Hole (mm)	Diameter × Height (mm)
Circumferential beam	30 × 10.489 × 8	t_1_ = 0.9945	
Radical beam	16 × 5.3 × 6	t_2_ = 0.5, t_3_ = 1, t_4_ = 1	
Central platform			54 × 6

**Table 3:** Plus or minus of strain under forces or torques

	R11	R12	R13	R14	R21	R22	R23	R24	R31	R32	R33	R34	R61	R62	R63	R64
*F_x_*	+	+	−	−	+	+	−	−	0	0	0	0	+	+	−	−
*F_y_*	0	0	0	0	0	0	0	0	+	+	−	−	−	+	−	+
*F_z_*	+	−	+	−	−	+	−	+	−	+	−	+	0	0	0	0
*M_x_*	0	0	0	0	0	0	0	0	−	+	+	−	0	0	0	0
*M_y_*	+	−	−	+	−	+	+	−	0	0	0	0	0	0	0	0
*M_z_*	0	0	0	0	0	0	0	0	0	0	0	0	−	+	+	−
	**R41**	**R41′**	**R42**	**R42′**	**R43**	**R43′**	**R44**	**R44′**	**R51**	**R51′**	**R52**	**R52′**	**R53**	**R53′**	**R54**	**R54′**
*F_x_*	+	+	−	−	−	−	+	+	+	−	−	+	−	+	+	−
*F_y_*	−	+	+	−	+	−	−	+	+	+	−	−	−	−	+	+
*F_z_*	+	+	−	−	+	+	−	−	+	+	−	−	+	+	−	−
*M_x_*	0	0	0	0	0	0	0	0	+	+	−	−	+	+	−	−
*M_y_*	+	+	−	−	+	+	−	−	0	0	0	0	0	0	0	0
*M_z_*	+	−	−	+	+	−	−	+	+	−	−	+	+	−	−	+

**Table 4:** Dimensions of two sensor models

	Main Beam Width × Height (mm)	Floating Beam Length × Width × Height (mm)	Central Platform Length × Width × Height (mm)	External Diameter (mm)	
Sensor 1	5 × 5	15.5 × 1 × 5	16 × 16 × 7	76	
	**Radial Beam Width × Height (mm)**	**Circumferential Beam Length × Width × Height (mm)**	**Central Platform Length × Width × Height (mm)**	**External Diameter (mm)**	**The Minimum Thickness Beside the Hole (mm)**
Sensor 2	5 × 5	15.5 × 9.4 × 5	16 × 16 × 7	76	t_1_ = 0.8, t_2_ = 0.5, t_3_ = 0.75, t_4_ = 0.75

**Table 5:** Relationship between total strain and loading force or torque

	*F_x_* = 100 N	*F_z_* = 100 N	*M_x_* = 3 N·m	*M_z_* = 3 N·m
Sensor 1	2.18 × 10^−3^	1.48 × 10^−3^	2.37 × 10^−3^	1.34 × 10^−3^
Sensor 2	2.25 × 10^−3^	2.78 × 10^−3^	2.26 × 10^−3^	2.38 × 10^−3^

**Table 6:** First six orders natural frequencies and the modes of vibration.

Characteristic of Vibration	Translation along X-axis	Translation along Y-axis	Translation along Z-axis	Rotation around X-axis	Rotation around Y-axis	Rotation around Z-axis
Frequency of sensor 1 (Hz)	1934.2	1934.3	2325.6	6913.8	6913.6	9075.5
Frequency of sensor 2 (Hz)	5910.9	5911.1	3742.5	11185.0	11,185.0	11,107.0
